# The Effect of Tinted Filters on the Visual Function of Patients with Retinitis Pigmentosa

**DOI:** 10.18502/jovr.v20.17489

**Published:** 2025-12-19

**Authors:** Alireza Riazifar, Ali Mirzajani, Hassan Hashemi, Alireza Jamali, Mehdi Khabazkhoob, Razieh Bahreini, Abbasali Yekta, Ebrahim Jafarzadehpur

**Affiliations:** ^1^Noor Research Center for Ophthalmic Epidemiology, Noor Eye Hospital, Tehran, Iran; ^2^Rehabilitation Research Center, Department of Optometry, School of Rehabilitation Sciences, Iran University of Medical Sciences, Tehran, Iran; ^3^Noor Ophthalmology Research Center, Noor Eye Hospital, Tehran, Iran; ^4^Department of Medical Surgical Nursing, School of Nursing and Midwifery, Shahid Beheshti University of Medical Sciences, Tehran, Iran; ^5^Pacific University College of Optometry, Forest Grove, Oregon, USA; ^6^Department of Optometry, School of Paramedical Sciences, Mashhad University of Medical Sciences, Mashhad, Iran

**Keywords:** Contrast Sensitivity, Retinitis Pigmentosa, Tinted Filters

## Abstract

**Purpose:**

To investigate the effect of tinted filters on contrast threshold (CT) and visual evoked potentials (VEPs) in patients with retinitis pigmentosa (RP).

**Methods:**

In this cross-sectional study, RP patients with best corrected visual acuity better than 1 log MAR were evaluated. The CT was measured using the Freiburg Acuity and Contrast Test, and pattern reversals were analyzed for both time and frequency components. The effects of gray (15% light transmission, 410 nm cutoff), brown (18% light transmission, 410 nm cutoff), and yellow (27% light transmission with a 470 nm cutoff) tinted filters were evaluated and compared.

**Results:**

Eighteen participants (mean age 32.78 ± 7.35 years; 72.2% male) were evaluated. The mean CT of Weber was 3.46 ± 3.2%. Yellow filters decreased CT (mean difference [MD]: –0.597%, *P *= 0.054). Brown filters did not produce a significant change in the CT (MD: 0.233%, *P* = 0.620), while the gray filters caused a significant increase in the CT (MD: 1.423%, *P* = 0.004). Mode frequency (Fmod) decreased with gray filters (MD: –2.33%, *P* = 0.004). The latency and amplitude of VEPs improved with yellow filters (*P* < 0.05) but worsened with gray filters (*P* < 0.05). Additionally, the spherical equivalent showed an inverse correlation with CT in patients with RP (*r* = –0.997, *P* < 0.001).

**Conclusion:**

Using tinted filters as a low-vision aid can modify contrast sensitivity and both frequency and time domains of VEPs in patients with RP. Yellow filters appear to improve visual function, whereas gray filters may worsen it.

##  INTRODUCTION

Retinitis pigmentosa (RP) is the most common form of retinal dystrophy, which typically presents bilaterally and causes progressive loss of vision.^[1,2,3]^ The disease initially damages rod photoreceptors, followed by cone involvement.^[4]^ As a result, patients develop night blindness, tunnel vision, and impaired contrast due to abnormalities in rod function and the magnocellular and parvocellular pathways.^[5,6]^ Fundus examination reveals bone spicule pigmentation, vascular narrowing, and optic disc pallor.

Patients with RP exhibit higher levels of photophobia, especially to short-wavelength light, as well as light scattering. These disturbances reduce visual quality and contrast sensitivity across a range of spatial frequencies, most prominently at low spatial frequencies and under dim illumination.^[7]^ The use of tinted filters as a contrast-enhancing technique can reduce recovery time following changes in light adaptation by regulating both the intensity and wavelength of light entering the eyes, thereby improving visual function.^[8]^ Coring Photochromic Filters (CPF) have been proposed as the gold standard for low vision rehabilitation.^[9]^ However, controversy remains regarding the optimal filter wavelength for RP patients given the heterogeneous nature of the disease. Various filters, such as orange,^[9]^ yellow,^[7]^ or red^[10]^ have been evaluated in different studies; yet uncertainty persists concerning the most effective type and intensity for different clinical situations.

Previous studies on the effectiveness of color filters in RP have largely relied on subjective assessments, such as contrast sensitivity, yielding inconsistent results.^[11,12]^ Objective tests, such as visual evoked potentials (VEPs), may provide a more reliable means of evaluating filter effects. Evidence suggests that pattern-reversal VEPs are altered in patients with RP,^[13]^ and this test can objectively quantify changes in contrast sensitivity.^[14]^

Given the conflicting findings in the literature, the present study evaluated the effect of three commonly used tinted filters on contrast threshold (CT), along with electrophysiological responses, in patients with RP. The filters were selected based on their potential clinical utility in patients with RP, and the study design incorporated substantial reductions in ambient light (approximately 70-85%) to assess the differential impact of each filter.

##  METHODS

### Participants

This cross-sectional study was conducted on 18 eyes from patients aged 23-49 years with RP, recruited from the Iran RP Center in 2020. The diagnosis of RP was established primarily on clinical grounds, including a history of night blindness, restricted visual fields (with an intact central visual field of at least 30º), and characteristic fundoscopic findings such as bone spicule pigmentation, vascular narrowing, and optic disc pallor.^[1]^ Inclusion criteria were: best-corrected visual acuity (BCVA) better than 1 LogMAR (logarithm of the minimum angle of resolution) in the randomly selected study eye, minimal or no cataract presence, and absence of clinically detectable macular cysts. Participants were fully informed about the study, and written informed consent was obtained. The study adhered to the tenets of the Declaration of Helsinki and was approved by the Ethics Committee of Iran University of Medical Sciences.

### Examinations

A comprehensive systemic and ocular history was obtained from all patients, and demographic data were recorded accurately. Optometric examinations were performed to determine the BCVA under standard conditions using the Huvitz HCP-7000. Refractive status was assessed with the Huvitz Auto Refractor HRK-8000A. A complete dilated fundus examination (using 1% tropicamide) was performed with the Hagg-Streit BM 900 LED slit lamp and the Volk +90 D non-contact slit-lamp lens to verify compliance with the inclusion criteria.

Three Cocoons Filters (Polare lens technology) were evaluated: (1) gray filter with 15% visible light transition and 410 nm UV cutoff; (2) brown filter with 18% visible light transition and 410 nm UV cutoff; and (3) yellow filter with 27% visible light transition and 470 nm UV cutoff. Changes in the CT, mode frequency (Fmod), amplitude, and the latency of the P100 wave of the pattern-reversal VEP with each filter were analyzed. Filter selection and density were based on the luminous efficiency function.^[15]^ Additionally, these filters are among the most commonly used filters in patients with retinal structural disorders.^[16]^ The optical density of the filters was chosen to evaluate changes in the intensity of light entering the eye, in addition to the chromatic properties of the filters.

### Contrast Threshold (CT) Evaluation

CT is defined as the lowest level of contrast required to detect a target. Contrast sensitivity is the inverse of the numerical value of the CT. In the present study, CT was measured based on the Weber criterion using the Freiburg Acuity and Contrast Test (FrACT). FrACT, a reliable test for measuring contrast sensitivity, was administered at a distance of 1.5 m, where one pixel on the screen corresponded to 0.57 arcminutes under standardized lighting conditions. The Landolt C optotype, presented in eight different directions, was displayed randomly three times for each filter to measure CT.^[17]^

Filters were mounted over the patients' corrective lenses using clips. To minimize afterimage effects, a 10-minute adaptation period was allowed between filter conditions. The order of filter application was randomized for each patient. Participants were instructed to identify the Landolt C optotypes, and their responses were recorded for all four conditions. All measurements were performed with undilated pupils.

### Visual Evoked Potential (VEP) Evaluation

VEPs were evaluated using the MetroVision electrodiagnostic system (MonoPack 1, Perenchies, France). Tests were performed under mesopic lighting and in accordance with ISCEV standards.^[18]^ No electronic devices were placed near the participants to avoid interference. Gold cup electrodes (ground, active, and reference) were positioned according to the international 10-20 system. Patients were seated one meter from the stimulus screen with refractive error fully corrected.

For optimal signal quality, the scalp surface was cleaned with alcohol, and electrodes filled with conductive gel were affixed using medical adhesive. Electrode-skin resistance was maintained below 50 Ω , with inter-electrode differences < 3 Ω . Binocular pattern-reversal VEPs were elicited using a 30-arcminute check size at high contrast, presented on a 19-inch monitor with a mean luminance of 50 cd/m². The mean luminance of the test room was 80 cd/m^2^. The stimuli were reversed at a temporal frequency of 1 Hz (two reversals per second).

P100 amplitude, latency time, and Fmod were measured under four randomized conditions: baseline (no filter), yellow filter, brown filter, and gray filter. To reduce carryover effects, a washout interval was provided between filter conditions. VEP recordings were performed one day after the initial examinations and CT testing. Each recording consisted of 100 sweeps averaged for reproducibility, and all waveforms were recorded twice.

The frequency-domain analysis was performed using the Welch power spectral density (PSD) of the VEP responses. Fmod was defined as the frequency corresponding to the peak of the PSD, representing the dominant frequency component of the signal.^[19]^ While time-domain VEP reflects changes in the signal over time, frequency-domain VEP demonstrates the distribution of power across frequencies. Assessing both domains allowed a more comprehensive evaluation of visual signal transmission.

### Sample Size

The sample size was estimated based on a study by Janaky et al,^[20]^ which reported a mean P100 latency of 136.5 ± 15.84 ms in patients with RP. Assuming an error margin of 7 ms, a significance level of 0.05, and a 10% dropout rate, a minimum of 22 participants was required.

### Statistical Analysis

Quantitative variables were presented as mean ± standard deviation. Normality of the distribution was evaluated using the Kolmogorov-Smirnov test. Differences among conditions were analyzed using repeated-measures ANOVA. Pairwise comparisons between each filter and baseline values were conducted with *t*-tests. Mean differences were calculated as the difference between baseline and post-filter values. Correlations between parameters were assessed using Pearson's or Spearman's correlation coefficients, depending on the normality of the distributions. A *P*-value < 0.05 was considered statistically significant. Statistical analyses were performed with SPSS software (version 26.0; SPSS Inc., Chicago, IL, USA).

**Table 1:** Mean and standard deviation of Weber contrast threshold with and without tinted filters

**Weber contrast threshold (%)**	**Mean ± SD (95% CI)**	**MD**	***P*** **-value^2^**
Without filter	3.46 ± 3.21 (1.85 to 5.05)	0	**–**
Yellow filter	2.86 ± 2.31 (1.70 to 4.01)	–0.597	0.054
Brown filter	3.69 ± 2.75 (2.31 to 5.05)	0.233	0.620
Gray filter	4.88 ± 3.76 (3.00 to 6.74)	1.423	0.004
*P*-value ${}^{1}$	< 0.001		
MD, mean difference between each measurement and baseline (without filter); *P*-value^1^, repeated-measures ANOVA, used to evaluate differences across all conditions; *P*-value^2^, Paired *t*-test, used to compare each filter condition with baseline (without filter); *P*-values < 0.05 were considered statistically significant.			

**Table 2:** Mean and standard deviation of VEP parameters in patients with retinitis pigmentosa, with and without filters

	**Amplitude (µv)**			**Latency (msec)**			**Fmod (Hz)**		
	**Mean ± SD (95% CI)**	**MD**	**P2**	**Mean ± SD (95% CI)**	**MD**	**P2**	**Mean ± SD (95% CI)**	**MD**	**P2**
Without filter	4.31 ± 2.61 (3.01 to 5.61)	0	–	119.0 ± 8.24 (114.9 to 123.1)	0	–	8.83 ± 3.58 (7.05 to 10.62)	0	–
Yellow filter	4.69 ± 2.80 (3.30 to 6.08)	0.382	0.001	115.4 ± 7.22 (111.7 to 118.9)	–3.611	0.001	10.11 ± 2.51 (8.86 to 11.36)	1.278	0.832
Brown filter	4.18 ± 2.54 (2.91 to 5.45)	–0.129	0.139	120.0 ± 7.42 (116.3 to 123.6)	1.000	0.195	7.50 ± 2.20 (6.40 to 8.60)	–1.333	0.750
Gray filter	4.07 ± 2.46 (2.84 to 5.29)	–0.243	0.012	121.0 ± 8.93 (116.5 to 125.4)	2.000	0.015	6.50 ± 1.94 (5.53 to 7.47)	–2.333	0.004
P1	< 0.001			< 0.001			< 0.001		
µv, microvolt; msec, millisecond; Hz, hertz; MD, mean difference between each measurement and baseline (without filter); P1, repeated-measures ANOVA, used to evaluate differences across all conditions; P2, paired *t*-test, used to compare each filter condition with baseline (without filter); *P*-values < 0.05 were considered statistically significant.									

##  RESULTS

A total of 22 participants were enrolled, but 4 were excluded for not cooperating with the study tests. Ultimately, 18 participants (mean age: 32.78 ± 7.35 years; 72.2% male [14 individuals]) were included in the analysis. The mean spherical equivalent (SE) and BCVA were –2.20 ± 4.04 D and 0.73 ± 0.26 LogMAR, respectively.

The mean CT of Weber was 3.46 ± 3.2%. The yellow filter decreased CT with borderline significance (MD: –0.597%, *P *= 0.054). The brown filter produced no significant change (MD: 0.233%, *P *= 0.620), whereas the gray filter significantly increased CT (MD: 1.423%, *P *= 0.004) [Table 1].

The mean Fmod at baseline was 8.83 ± 3.58 Hz, which significantly decreased with the gray filter (6.50 ± 1.94 Hz, *P *=0.004). The yellow and brown filters did not significantly affect the Fmod (*P* > 0.05). P100 amplitude increased with the yellow filter (4.31 ± 2.61 to 4.69 ± 2.80 µv, *P *= 0.001) and decreased with the gray filter (4.07 ± 2.46 µv, *P *= 0.012). Latency was reduced with the yellow filter (MD: –3.611 ms, *P *= 0.001), but increased with the gray filter (MD: 2.000 ms, *P *= 0.015). These electrophysiological changes are summarized in Table 2.

Correlation analysis revealed that SE was inversely correlated with CT in patients with RP (*r* = –0.997, *P* < 0.001). This correlation persisted with color filters, with the strongest correlation observed for the yellow filter (*r* = –0.904, *P* < 0.001; brown filter: *r* = –0.661, *P *= 0.003; gray filter: *r* = –0.755, *P* < 0.001).

##  DISCUSSION

This study evaluated the effect of tinted filters on changes in CT and VEP in both frequency and time domains in patients with RP. In individuals with restricted visual fields, conventional low-vision aids such as telescopes and microscopes provide limited benefit.^[21]^ In contrast, tinted filters not only protect ocular tissues from blue-light hazards but also reduce chromatic aberration, Rayleigh scatter, UV-induced lenticular fluorescein, and rod-cell oversaturation, thereby enhancing visual function by selectively controlling the light wavelength and intensity.^[7,22]^

In our study, the yellow filter showed a borderline improvement in CT. Given the relatively low BCVA of participants (0.73 ± 0.26 LogMAR), even modest changes in CT may contribute to functional improvements. Several prior studies support the beneficial effect of yellow or short-, wavelength-absorbing filters in RP.^[7,23,24]^ Cedrum-Sanchez et al demonstrated that filters with a 450 nm cutoff improved visual discrimination under dim illumination in RP patients by reducing diffraction, scatter, and aberrations.^[7]^ Wetzel et al also reported significant improvements in CS with customized cutoff filters.^[24]^ However, other studies suggest that tinted filters are more effective in patients with anterior scatter than in RP or diabetic patients,^[25]^ findings that conflict with our results and those of Sadeghpour et al.^[25,26]^ Morrissette et al reported that users of a CPF 550 nm filter as a low-vision aid experienced reduced adaptation time to changes in illumination, along with improved visual functioning and ocular comfort.^[27]^ In the study by Eperjesi et al, RP patients were excluded due to the progressive nature of their condition;^[9]^ whereas our study demonstrates reliable outcomes for this group.

The observed differences can be explained by Carracedo et al's findings, who reported that the use of the CPF 527 filter (orange range) increases CS in RP patients, both with and without glare, with the greatest effect observed at a spatial frequency of 3 CPD and no effect at 12 CPD. Additionally, based on subjective reports and questionnaire responses, the subjects expressed greater satisfaction when using the filter.^[16]^ Importantly, the transmitted spectrum and the specific test used to assess CS can strongly influence outcomes, as different tests evaluate different ranges of spatial frequencies.

Variability in outcomes may be attributed to differences in test methodology, transmitted spectrum, spatial frequency range, and patient age. For instance, Mahjoob et al reported greater improvements with yellow filters in elderly patients with low vision than in the younger ones.^[28]^ Similarly, ambient lighting has been shown to influence filter efficacy. Abraham et al observed that yellow filters had maximal benefit at 100 lux in patients with maculopathy, while effectiveness in cataract varied with brightness and cataract severity.^[8]^ Filter density also plays a role, with Kalikivayi et al noting that yellow and pink filters improved CS by up to 60% at low densities but worsened it at higher densities.^[29]^

Based on the findings of the current study, yellow filters also increased the amplitude and reduced latency without a significant change in Fmod. Paranhos et al^[31]^ demonstrated a reduction in P100 wave amplitude in patients with RP compared to normal subjects, which is likely related to changes in the parvocellular and magnocellular pathways as well as higher-order visual pathways.^[6]^ Cellular evaluations have also shown that S-cones in patients with RP are more severely affected than L- and M-cones.^[35]^ Therefore, it appears that filtering short wavelengths may reduce the activity of S-cone cells, thereby improving visual function.^[32]^

Since the Fmod component reflects information transformed into the frequency domain for processing in the brain,^[19]^ the absence of a significant difference in this domain, combined with positive changes in the temporal components of the VEP, suggests that while the use of a yellow filter in patients with RP does not alter the transmitted information, it may help modulate negative lateral components and thereby enhance the speed of information transmission to the visual cortex. This effect, together with the borderline improvement observed in CS, could contribute to better visual performance in patients with RP. Nevertheless, factors such as variations in frequency content, filter density, lighting conditions, and the severity of RP may influence the effectiveness of the intervention. These variables should therefore be carefully considered in clinical decision-making to optimize patient outcomes.

In contrast, brown filters had no significant effect on CT or electrophysiological parameters in our study. As filters with the highest absorption below 550 nm showed the greatest CS improvements,^[33]^ the higher absorption range of brown filters (up to ∼ 600 nm) may explain the limited efficacy.^[34]^ This shows that the wavelengths in the range of approximately 500-600 nm play a beneficial role in the visual performance of individuals with RP, and further investigation of this effect is recommended in future studies. Electrophysiological evaluations in the present studies also indicate that the use of the brown filter does not affect either the time or frequency domains of VEP.

Interference with color perception is one of the major limitations of color filters.^[35]^ Although filters such as EnChroma have been shown to enhance color discrimination in people with color vision deficiencies,^[36]^ their use in those without such defects may cause disturbances in color perception. Based on this concern, Orlance et al recommended red filters for patients with RP and light sensitivity due to their protective effect on disease progression. However, this tint has been reported to induce Tritan-like color vision abnormalities, disturb sleep, and exacerbate night vision.^[10]^ Therefore, a comprehensive evaluation of different aspects of the visual system is essential when prescribing filters. Notably, Orlance et al also indicated that yellow filters with a 470 nm cut-off do not produce any of these adverse effects.^[10]^

Fez et al showed that while the use of color filters may improve CS, it also introduces errors in color discrimination, whereas the use of gray filters did not affect discrimination.^[37]^ In contrast, the results of the present study show that gray filters significantly reduce CS in patients with RP, thereby greatly diminishing both the quality of vision and life. Electrophysiological evaluations further revealed that gray filters reduce Fmod, increase latency, and also decrease the amplitude of the P100 wave. Mahjoob et al found that gray filters in healthy individuals increase the CS in high light intensity (54000 lux, equivalent to sunlight exposure).^[38]^ Conversely, Bailie et al indicate that in age-related macular degeneration, neutral density filters reduce both CS and visual acuity.^[39]^ Similarly, Huchzermeyer et al observed that in occult macular dystrophy associated with RP1L1, perifoveal L- and M-cone function is reduced and remains dependent on intact rod cell activity.^[40]^ Taken together, these findings suggest that differences in cone and rod cell function in patients with RP may shift the spectral balance toward rod cell activity, thereby producing variable outcomes in visual function, CS, and electrophysiological responses. Further research is needed to clarify these mechanisms.

The results of the present study demonstrated an inverse correlation between CT and SE. Stoimenova reported that the CS of patients with myopia is lower than that of emmetropic ones, and that CS decreases with increasing degrees of myopia.^[41]^ In contrast, Li et al found that CS at low spatial frequencies in low and moderate myopia is higher than in other types of refractive error; however, with increasing spatial frequency and the overall decrease in CS across all refractive errors, myopia was associated with higher CS than other refractive errors at high spatial frequencies.^[42]^ Karatepe et al also noted that hyperopia is associated with the lowest CS among refractive errors.^[43]^ Consistent with these findings, our results indicate that an increase in hyperopia (or a reduction in myopia) is negatively associated with contrast sensitivity.

In summary, this study showed that tinted filters may serve as effective low-vision aids for patients with RP, as they can modify both contrast sensitivity and electrophysiological responses. Specifically, yellow filters appear beneficial in improving visual quality, whereas gray filters should be avoided in these individuals.

##  Financial Support and Sponsorship

None.

##  Conflicts of Interest

None.

## References

[B1] Parmeggiani F Clinics, epidemiology and genetics of retinitis pigmentosa. Curr Genomics 2011;12:236–237. 22131868 10.2174/138920211795860080 PMC3131730

[B2] Mishra C Sen S Kannan NB Ramasamy K Unilateral retinitis pigmentosa associated with cystoid macular oedema. Natl Med J India 2023;36. 10.25259/NMJI_651_20 38692617

[B3] O'Neal TB Tripathy K Luther EE Retinitis pigmentosa. StatPearls Publishing; 2024. 30137803

[B4] Nguyen XT Moekotte L Plomp AS Bergen AA van Genderen MM Boon CJ Retinitis pigmentosa: Current clinical management and emerging therapies. Int J Mol Sci 2023;24:7481. 37108642 10.3390/ijms24087481 PMC10139437

[B5] Hartong DT Berson EL Dryja TP Retinitis pigmentosa. Lancet 2006;368:1795–1809. 17113430 10.1016/S0140-6736(06)69740-7

[B6] Alexander KR Rajagopalan AS Seiple W Zemon VM Fishman GA Contrast response properties of magnocellular and parvocellular pathways in retinitis pigmentosa assessed by the visual evoked potential. Invest Ophthalmol Vis Sci 2005;46:2967–2973. 16043873 10.1167/iovs.05-0231 PMC1283742

[B7] Cedrún-Sánchez JE Chamorro E Bonnin-Arias C Aguirre-Vilacoro V Castro JJ Sánchez-Ramos C Visual discrimination increase by yellow filters in Retinitis pigmentosa. Optom Vis Sci 2016;93:1537–1544. 27391534 10.1097/OPX.0000000000000924

[B8] Abraham CH Morny E Aboagye-MacCarthy A Ocansey S Ntodie M Sakyi-Badu G The effect of filters and varying illumination on contrast sensitivity in eyes with moderate to severe visual impairment. Int Ophthalmol 2023;43:3329–3337. 37193933 10.1007/s10792-023-02738-7

[B9] Eperjesi F Fowler CW Evans BJ Do tinted lenses or filters improve visual performance in low vision? A review of the literature. Ophthalmic Physiol Opt 2002;22:68–77. 11829009 10.1046/j.1475-1313.2002.00004.x

[B10] Orlans HO Merrill J Barnard AR Charbel Issa P Peirson SN MacLaren RE Filtration of short-wavelength light provides therapeutic benefit in retinitis pigmentosa caused by a common rhodopsin mutation. Invest Ophthalmol Vis Sci 2019;60:2733–2742. 31247114 10.1167/iovs.19-26964

[B11] Mahjoob M Heydarian S Effects of color filters and anti-reflective coating on contrast sensitivity under glare condition. J Res Clin Med 2020;8:28.

[B12] Mahjoub M Shandiz JH Validad MH Moghadam HM Tayebeh HK Abadi RT The effect of color filters on the visual acuity and contrast sensitivity in low vision patients. Majallah-i Danishgah-i Ulum-i Pizishki-i Babul 2010;11:53–57.

[B13] Papathanasopoulos PG Papakostopoulos D Pattern reversal visual evoked potentials in retinitis pigmentosa. Int J Psychophysiol 1994;16:245–250. 8089043 10.1016/0167-8760(89)90051-2

[B14] Kothari R Bokariya P Singh S Singh R A comprehensive review on methodologies employed for visual evoked potentials. Scientifica 2016;2016:9852194. 27034907 10.1155/2016/9852194 PMC4789528

[B15] Sharpe LT Stockman A Jagla W Jägle H A luminous efficiency function, V*(λ), for daylight adaptation. J Vis 2005;5:948–968. 16441195 10.1167/5.11.3

[B16] Carracedo G Carballo J Loma E Felipe G Cacho I Contrast sensitivity evaluation with filter contact lenses in patients with retinitis pigmentosa: A pilot study. J Optom 2011;4:134–139.

[B17] Mohammadi A Hashemi H Mirzajani A Yekta A Jafarzadehpur E Khabazkhoob M Comparison of two methods for measuring contrast sensitivity in anisometropic amblyopia. J Curr Ophthalmol 2018;30:343–347. 30555968 10.1016/j.joco.2018.04.002 PMC6276632

[B18] Odom JV Bach M Brigell M Holder GE McCulloch DL Mizota A International Society for Clinical Electrophysiology of Vision. ISCEV standard for clinical visual evoked potentials: (2016 update). Doc Ophthalmol 2016;133:1–9. 10.1007/s10633-016-9553-y 27443562

[B19] Hassankarimi H Jafarzadehpur E Mohammadi A Noori SM Low-contrast pattern-reversal visual evoked potential in different spatial frequencies. J Ophthalmic Vis Res 2020;15:362–371. 32864067 10.18502/jovr.v15i3.7455 PMC7431726

[B20] Janáky M Pálffy A Horváth G Tuboly G Benedek G Pattern-reversal electroretinograms and visual evoked potentials in retinitis pigmentosa. Doc Ophthalmol 2008;117:27–36. 18034272 10.1007/s10633-007-9099-0

[B21] Krefman RA Reversed telescopes on visual efficiency scores in field-restricted patients. Am J Optom Physiol Opt 1981;58:159–162. 7223844 10.1097/00006324-198102000-00008

[B22] Van den Berg T Red glasses and visual function in retinitis pigmentosa. Documenta Ophthalmologica 1989;73:255-274. 2638244 10.1007/BF00155095

[B23] Ahmad A Sughra U Habib M Imran MA Contrast sensitivity improvement with yellow filter in low vision patients. ISRA Med J 2018;9:402–405.

[B24] Wetzel C Auffarth GU Krastel H Blankenagel A Alexandridis E [Improving contrast sensitivity by cut-off filters in high adaptation luminance levels in retinitis pigmentosa]. Ophthalmologe 1996;93:456–462. 8963147

[B25] Leat SJ North RV Bryson H Do long wavelength pass filters improve low vision performance? Ophthalmic Physiol Opt 1990;10:219–224. 2216468

[B26] Sadeghpour N Alishiri AA Ajudani R Khosravi MH Amiri MA Sadeghpour O Quantity and quality of vision using tinted filters in patients with low vision due to diabetic retinopathy. J Ophthalmic Vis Res 2015;10:429–432. 27051488 10.4103/2008-322X.158893 PMC4795393

[B27] Morrissette DL Mehr EB Keswick CW Lee PN Users’ and nonusers’ evaluations of the CPF 550 lenses. Am J Optom Physiol Opt 1984;61:704–710. 6517129

[B28] Mahjoob M Heydarian S Koochi S Effect of yellow filter on visual acuity and contrast sensitivity under glare condition among different age groups. Int Ophthalmol 2016;36:509–514. 26613932 10.1007/s10792-015-0154-7

[B29] Kalikivayi V Kalikivayi L Influence of various densities of yellow and pink tinted spectacles on contrast sensitivity. Clin Exp Ophthalmol 2021;1.

[B30] Sharma R Joshi S Singh KD Kumar A Visual evoked potentials: Normative values and gender differences. J Clin Diagn Res 2015;9:CC12–CC15. 10.7860/JCDR/2015/12764.6181 PMC4572953 26393122

[B31] Paranhos FR Katsum O Arai M Nehemy MB Hirose T Pattern reversal visual evoked response in retinitis pigmentosa. Doc Ophthalmol 1998–1999;96:321–331. 10.1023/a:1001853420082 10855808

[B32] Swanson WH Birch DG Anderson JL S-cone function in patients with retinitis pigmentosa. Invest Ophthalmol Vis Sci 1993;34:3045–3055. 8407212

[B33] Leat SJ North RV Bryson H Do long wavelength pass filters improve low vision performance? Ophthalmic Physiol Opt 1990;10:219–224. 2216468

[B34] Phillips SM Smith GD Light absorption by charge transfer complexes in brown carbon aerosols. Environ Sci Technol Lett 2014;1:382–386.

[B35] Álvaro L Linhares JM Formankiewicz MA Waugh SJ Coloured filters can simulate colour deficiency in normal vision but cannot compensate for congenital colour vision deficiency. Sci Rep 2022;12:11140. 35778454 10.1038/s41598-022-13877-9 PMC9249763

[B36] Bastien K Mallet D Saint-Amour D Characterizing the effects of enchroma glasses on color discrimination. Optom Vis Sci 2020;97:903–910. 33055508 10.1097/OPX.0000000000001581

[B37] de Fez MD Luque MJ Viqueira V Enhancement of contrast sensitivity and losses of chromatic discrimination with tinted lenses. Optom Vis Sci 2002;79:590–597. 12322929 10.1097/00006324-200209000-00010

[B38] Mahjoob M Azimi A Heravian J Momeni Moghadam H Mahjoob F The effect of various colors of sunglasses in visual function. Majallah-i Danishgah-i Ulum-i Pizishki-i Kirmanshah 2012;16:e78875.

[B39] Bailie M Wolffsohn JS Stevenson M Jackson AJ Functional and perceived benefits of wearing coloured filters by patients with age-related macular degeneration. Clin Exp Optom 2013;96:450–454. 23472744 10.1111/cxo.12031

[B40] Huchzermeyer C Fars J Kremers J Kühlewein L Kempf M Ott S Photoreceptor-specific temporal contrast sensitivities in RP1L1-associated occult macular dystrophy. Invest Ophthalmol Vis Sci 2023;64:33. 10.1167/iovs.64.7.33 PMC10289274 37342031

[B41] Stoimenova BD The effect of myopia on contrast thresholds. Invest Ophthalmol Vis Sci 2007;48:2371–2374. 17460304 10.1167/iovs.05-1377

[B42] Li Z Hu Y Yu H Li J Yang X Effect of age and refractive error on quick contrast sensitivity function in Chinese adults: A pilot study. Eye 2021;35:966–972. 32518399 10.1038/s41433-020-1009-7 PMC8026637

[B43] Karatepe AS Köse S Eğrilmez S Factors affecting contrast sensitivity in healthy individuals: A pilot study. Turk J Ophthalmol 2017;47:80–84. 28405481 10.4274/tjo.93763 PMC5384124

